# Supplementary material for: Time-series transcriptome analysis identified differentially expressed genes in broiler chicken infected with mixed Eimeria species
Source: Front Genet. 2022 Aug 8;13:886781. doi: 10.3389/fgene.2022.886781 (PMC9393255; doi:10.3389/fgene.2022.886781)
Supplement: Supplementary file 2 [file DataSheet1.ZIP › 7dpi_GO.Gsea.1625106495923/GOBP_REGULATION_OF_RAS_PROTEIN_SIGNAL_TRANSDUCTION.html]

Details for gene set GOBP\_REGULATION\_OF\_RAS\_PROTEIN\_SIGNAL\_TRANSDUCTION[GSEA]

|  || Dataset | TMM\_7dpi\_gct\_format\_7dpi\_gct\_format.Class\_4dpi.cls #PC\_versus\_NC.Class\_4dpi.cls #PC\_versus\_NC\_repos |
| Phenotype | Class\_4dpi.cls#PC\_versus\_NC\_repos |
| Upregulated in class | 0 |
| GeneSet | GOBP\_REGULATION\_OF\_RAS\_PROTEIN\_SIGNAL\_TRANSDUCTION |
| Enrichment Score (ES) | -0.49603927 |
| Normalized Enrichment Score (NES) | -1.8407073 |
| Nominal p-value | 0.0 |
| FDR q-value | 0.0941251 |
| FWER p-Value | 0.8064 |
Table: GSEA Results Summary

  

Fig 1: Enrichment plot: GOBP\_REGULATION\_OF\_RAS\_PROTEIN\_SIGNAL\_TRANSDUCTION      
 Profile of the Running ES Score & Positions of GeneSet Members on the Rank Ordered List

  

| SYMBOL | TITLE | RANK IN GENE LIST | RANK METRIC SCORE | RUNNING ES | CORE ENRICHMENT || 1 | SQSTM1 | na | 594 | 0.688 | -0.0412 | No |
| 2 | ARHGAP44 | na | 694 | 0.639 | -0.0413 | No |
| 3 | RALGPS2 | na | 696 | 0.638 | -0.0331 | No |
| 4 | FLOT1 | na | 969 | 0.532 | -0.0491 | No |
| 5 | RASAL1 | na | 1275 | 0.434 | -0.0692 | No |
| 6 | HRAS | na | 1342 | 0.420 | -0.0694 | No |
| 7 | CDC42SE1 | na | 1568 | 0.362 | -0.0837 | No |
| 8 | TGFB2 | na | 1954 | 0.278 | -0.1125 | No |
| 9 | ITGA3 | na | 2012 | 0.270 | -0.1138 | No |
| 10 | RASA4B | na | 2247 | 0.230 | -0.1306 | No |
| 11 | CCDC125 | na | 2479 | 0.195 | -0.1475 | No |
| 12 | NET1 | na | 2635 | 0.172 | -0.1584 | No |
| 13 | LPAR6 | na | 2712 | 0.162 | -0.1627 | No |
| 14 | DYNLT1 | na | 2885 | 0.140 | -0.1754 | No |
| 15 | PLCE1 | na | 2983 | 0.129 | -0.1819 | No |
| 16 | RAC1 | na | 2998 | 0.126 | -0.1814 | No |
| 17 | MET | na | 3037 | 0.122 | -0.1831 | No |
| 18 | ARHGDIA | na | 3051 | 0.120 | -0.1826 | No |
| 19 | ARHGAP24 | na | 3334 | 0.083 | -0.2053 | No |
| 20 | PSD2 | na | 3404 | 0.074 | -0.2102 | No |
| 21 | TIMP2 | na | 3439 | 0.069 | -0.2121 | No |
| 22 | OPHN1 | na | 3542 | 0.059 | -0.2200 | No |
| 23 | ARHGDIB | na | 3731 | 0.037 | -0.2353 | No |
| 24 | P2RY8 | na | 3953 | 0.010 | -0.2538 | No |
| 25 | RASGRF2 | na | 3967 | 0.008 | -0.2548 | No |
| 26 | CUL3 | na | 4090 | -0.005 | -0.2650 | No |
| 27 | MYOC | na | 4194 | -0.016 | -0.2735 | No |
| 28 | LRRC59 | na | 4425 | -0.042 | -0.2924 | No |
| 29 | NOTCH1 | na | 4525 | -0.053 | -0.3000 | No |
| 30 | LPAR1 | na | 4729 | -0.074 | -0.3162 | No |
| 31 | GPR20 | na | 4920 | -0.095 | -0.3310 | No |
| 32 | PSD | na | 5025 | -0.106 | -0.3384 | No |
| 33 | DLC1 | na | 5074 | -0.110 | -0.3410 | No |
| 34 | RAF1 | na | 5142 | -0.116 | -0.3451 | No |
| 35 | SHOC2 | na | 5171 | -0.118 | -0.3460 | No |
| 36 | LPAR4 | na | 5198 | -0.121 | -0.3466 | No |
| 37 | CSF1 | na | 5321 | -0.133 | -0.3552 | No |
| 38 | APOA1 | na | 5412 | -0.141 | -0.3609 | No |
| 39 | NUP62 | na | 5756 | -0.176 | -0.3876 | No |
| 40 | SHC1 | na | 5796 | -0.179 | -0.3885 | No |
| 41 | PPP2CB | na | 6246 | -0.229 | -0.4234 | No |
| 42 | GPR35 | na | 6280 | -0.233 | -0.4232 | No |
| 43 | MAPKAP1 | na | 6306 | -0.235 | -0.4223 | No |
| 44 | KANK1 | na | 6338 | -0.238 | -0.4218 | No |
| 45 | RABL3 | na | 6340 | -0.238 | -0.4188 | No |
| 46 | DENND1A | na | 6442 | -0.248 | -0.4241 | No |
| 47 | STARD13 | na | 6491 | -0.253 | -0.4249 | No |
| 48 | CYTH1 | na | 6504 | -0.254 | -0.4226 | No |
| 49 | ABL1 | na | 6549 | -0.259 | -0.4230 | No |
| 50 | ARHGAP17 | na | 6614 | -0.267 | -0.4249 | No |
| 51 | GRB2 | na | 6686 | -0.274 | -0.4274 | No |
| 52 | STAMBP | na | 6721 | -0.277 | -0.4267 | No |
| 53 | MAPRE2 | na | 6766 | -0.281 | -0.4267 | No |
| 54 | ARHGAP42 | na | 6782 | -0.284 | -0.4243 | No |
| 55 | IRS2 | na | 6867 | -0.293 | -0.4276 | No |
| 56 | CRK | na | 6900 | -0.297 | -0.4265 | No |
| 57 | FLCN | na | 6949 | -0.302 | -0.4266 | No |
| 58 | RASAL3 | na | 7020 | -0.311 | -0.4285 | No |
| 59 | PRAG1 | na | 7024 | -0.311 | -0.4248 | No |
| 60 | COL3A1 | na | 7109 | -0.320 | -0.4277 | No |
| 61 | CRKL | na | 7144 | -0.324 | -0.4264 | No |
| 62 | RAPGEF1 | na | 7164 | -0.325 | -0.4238 | No |
| 63 | KRAS | na | 7206 | -0.330 | -0.4230 | No |
| 64 | GPR4 | na | 7263 | -0.337 | -0.4233 | No |
| 65 | ROBO1 | na | 7316 | -0.343 | -0.4233 | No |
| 66 | F2RL3 | na | 7471 | -0.358 | -0.4316 | No |
| 67 | ARHGEF10 | na | 7521 | -0.363 | -0.4311 | No |
| 68 | RASGEF1A | na | 7529 | -0.365 | -0.4270 | No |
| 69 | ARHGAP35 | na | 7860 | -0.402 | -0.4496 | No |
| 70 | ARFGEF1 | na | 8006 | -0.421 | -0.4563 | No |
| 71 | PIK3CG | na | 8478 | -0.481 | -0.4898 | Yes |
| 72 | SPRY1 | na | 8482 | -0.481 | -0.4839 | Yes |
| 73 | SGSM3 | na | 8506 | -0.484 | -0.4795 | Yes |
| 74 | BCR | na | 8519 | -0.485 | -0.4743 | Yes |
| 75 | SPRY2 | na | 8604 | -0.500 | -0.4749 | Yes |
| 76 | PDGFRB | na | 8726 | -0.517 | -0.4784 | Yes |
| 77 | RASA3 | na | 8763 | -0.521 | -0.4747 | Yes |
| 78 | FBP1 | na | 8774 | -0.522 | -0.4688 | Yes |
| 79 | GBF1 | na | 8800 | -0.525 | -0.4641 | Yes |
| 80 | P2RY10 | na | 8840 | -0.529 | -0.4606 | Yes |
| 81 | SCAI | na | 8905 | -0.537 | -0.4591 | Yes |
| 82 | ARFGAP1 | na | 8953 | -0.542 | -0.4560 | Yes |
| 83 | ADGRG1 | na | 9008 | -0.550 | -0.4535 | Yes |
| 84 | ARF6 | na | 9017 | -0.550 | -0.4470 | Yes |
| 85 | CDC42SE2 | na | 9186 | -0.580 | -0.4537 | Yes |
| 86 | ABCA1 | na | 9260 | -0.592 | -0.4522 | Yes |
| 87 | SSX2IP | na | 9336 | -0.604 | -0.4507 | Yes |
| 88 | F2RL2 | na | 9360 | -0.608 | -0.4448 | Yes |
| 89 | EPS8 | na | 9414 | -0.617 | -0.4413 | Yes |
| 90 | NRP1 | na | 9452 | -0.624 | -0.4363 | Yes |
| 91 | RASGRP1 | na | 9465 | -0.626 | -0.4293 | Yes |
| 92 | LZTR1 | na | 9500 | -0.631 | -0.4240 | Yes |
| 93 | RASA2 | na | 9502 | -0.632 | -0.4159 | Yes |
| 94 | MAP4K4 | na | 9524 | -0.635 | -0.4094 | Yes |
| 95 | F2RL1 | na | 9532 | -0.636 | -0.4018 | Yes |
| 96 | ARHGEF18 | na | 9548 | -0.638 | -0.3948 | Yes |
| 97 | STMN3 | na | 9624 | -0.649 | -0.3928 | Yes |
| 98 | STMN1 | na | 9701 | -0.661 | -0.3906 | Yes |
| 99 | RDX | na | 9709 | -0.662 | -0.3827 | Yes |
| 100 | TNFAIP1 | na | 9717 | -0.663 | -0.3747 | Yes |
| 101 | ERBIN | na | 9756 | -0.667 | -0.3693 | Yes |
| 102 | KCTD10 | na | 9757 | -0.667 | -0.3607 | Yes |
| 103 | FBXO8 | na | 9846 | -0.681 | -0.3593 | Yes |
| 104 | DENND4C | na | 10096 | -0.727 | -0.3708 | Yes |
| 105 | IQSEC1 | na | 10136 | -0.738 | -0.3646 | Yes |
| 106 | RASGRF1 | na | 10171 | -0.746 | -0.3578 | Yes |
| 107 | ALS2 | na | 10208 | -0.751 | -0.3512 | Yes |
| 108 | MYO9B | na | 10222 | -0.753 | -0.3425 | Yes |
| 109 | HEG1 | na | 10291 | -0.769 | -0.3383 | Yes |
| 110 | CYTH3 | na | 10319 | -0.774 | -0.3306 | Yes |
| 111 | PIK3CB | na | 10335 | -0.777 | -0.3218 | Yes |
| 112 | DAB2IP | na | 10375 | -0.784 | -0.3150 | Yes |
| 113 | BCL6 | na | 10452 | -0.801 | -0.3110 | Yes |
| 114 | OGT | na | 10479 | -0.805 | -0.3028 | Yes |
| 115 | AKAP13 | na | 10490 | -0.807 | -0.2932 | Yes |
| 116 | DENND4A | na | 10574 | -0.827 | -0.2895 | Yes |
| 117 | NOTCH2 | na | 10580 | -0.828 | -0.2793 | Yes |
| 118 | ARHGEF28 | na | 10613 | -0.836 | -0.2711 | Yes |
| 119 | MFN2 | na | 10650 | -0.843 | -0.2633 | Yes |
| 120 | EPHB2 | na | 10703 | -0.854 | -0.2566 | Yes |
| 121 | SYNGAP1 | na | 10753 | -0.867 | -0.2496 | Yes |
| 122 | RALGPS1 | na | 10768 | -0.870 | -0.2395 | Yes |
| 123 | GPR174 | na | 10813 | -0.881 | -0.2318 | Yes |
| 124 | DENND3 | na | 10815 | -0.881 | -0.2205 | Yes |
| 125 | SH3BP1 | na | 10832 | -0.888 | -0.2104 | Yes |
| 126 | ARFGEF2 | na | 10852 | -0.894 | -0.2004 | Yes |
| 127 | FGF10 | na | 10866 | -0.897 | -0.1899 | Yes |
| 128 | KITLG | na | 10999 | -0.936 | -0.1890 | Yes |
| 129 | RASA1 | na | 11049 | -0.950 | -0.1808 | Yes |
| 130 | JAK2 | na | 11123 | -0.971 | -0.1744 | Yes |
| 131 | GPR65 | na | 11148 | -0.979 | -0.1638 | Yes |
| 132 | GPR18 | na | 11177 | -0.992 | -0.1533 | Yes |
| 133 | CYTH4 | na | 11221 | -1.006 | -0.1439 | Yes |
| 134 | LYN | na | 11314 | -1.043 | -0.1382 | Yes |
| 135 | ABL2 | na | 11336 | -1.051 | -0.1264 | Yes |
| 136 | FOXM1 | na | 11360 | -1.063 | -0.1146 | Yes |
| 137 | ARHGEF3 | na | 11458 | -1.101 | -0.1086 | Yes |
| 138 | SPRY3 | na | 11641 | -1.197 | -0.1084 | Yes |
| 139 | MADD | na | 11657 | -1.205 | -0.0941 | Yes |
| 140 | F2R | na | 11661 | -1.208 | -0.0788 | Yes |
| 141 | GPR55 | na | 11797 | -1.330 | -0.0730 | Yes |
| 142 | AUTS2 | na | 11812 | -1.347 | -0.0567 | Yes |
| 143 | ARFGEF3 | na | 11839 | -1.392 | -0.0409 | Yes |
| 144 | KIF14 | na | 11840 | -1.393 | -0.0229 | Yes |
| 145 | IGF1 | na | 11859 | -1.426 | -0.0060 | Yes |
| 146 | CBL | na | 11873 | -1.446 | 0.0115 | Yes |
Table: GSEA details [plain text format]

  

Fig 2: GOBP\_REGULATION\_OF\_RAS\_PROTEIN\_SIGNAL\_TRANSDUCTION      
 Blue-Pink O' Gram in the Space of the Analyzed GeneSet

  

Fig 3: GOBP\_REGULATION\_OF\_RAS\_PROTEIN\_SIGNAL\_TRANSDUCTION: Random ES distribution      
 Gene set null distribution of ES for **GOBP\_REGULATION\_OF\_RAS\_PROTEIN\_SIGNAL\_TRANSDUCTION**
